# Short-term effect of fine particulate matter and ozone on non-accidental mortality and respiratory mortality in Lishui district, China

**DOI:** 10.1186/s12889-021-11713-9

**Published:** 2021-09-13

**Authors:** Yuqi Chen, Zhigang Jiao, Ping Chen, Lijun Fan, Xudan Zhou, Yuepu Pu, Wei Du, Lihong Yin

**Affiliations:** 1 Key Laboratory of Environmental Medicine Engineering, Ministry of Education, School of Public Health, Southeast University, Nanjing, 210009 China; 2 Lishui Smart City Operating Command Center, Lishui, 211200 China

**Keywords:** Air pollution, O_3_, PM_2.5_, Generalized additive model, Mortality

## Abstract

**Background:**

In recent years, air pollution has become an imminent problem in China. Few studies have investigated the impact of air pollution on the mortality of the middle-aged and elderly people. Therefore, this study aims to evaluate the impact of PM_2.5_ (fine particulate matter) and O_3_ (ozone) on non-accidental mortality and respiratory mortality of the middle-aged and elderly people in Lishui District of Nanjing and provide the evidence for potential prevention and control measures of air pollution.

**Method:**

Using daily mortality and atmospheric monitoring data from 2015 to 2019, we applied a generalized additive model with time-series analysis to evaluate the association of PM_2.5_ and O_3_ exposure with daily non-accidental mortality and respiratory mortality in Lishui District. Using the population attributable fractions to estimate the death burden caused by short-term exposure to O_3_ and PM_2.5。_.

**Result:**

For every 10 μg/m^3^ increase in PM_2.5_, non-accidental mortality increased 0.94% with 95% confidence interval (CI) between 0.05 and 1.83%, and PM_2.5_ had a more profound impact on females than males. For every 10 μg/m^3^ increase in O_3_, respiratory mortality increased 1.35% (95% CI: 0.05, 2.66%) and O_3_ had a more profound impact on males than females. Compared with the single pollutant model, impact of the two-pollutant model on non-accidental mortality and respiratory mortality slightly decreased. In summer and winter as opposed to the other seasons, O_3_ had a more obvious impact on non-accidental mortality. The population attributable fractions of non-accidental mortality were 0.84% (95% CI:0.00, 1.63%) for PM_2.5_ and respiratory mortality were 0.14% (95% CI:0.01, 0.26%) for O_3_. For every 10 μg/m^3^ decrease in PM_2.5,_ 122 (95% CI: 6, 237) non-accidental deaths could be avoided. For every 10 μg/m^3^ decrease in O_3_, 10 (95% CI: 1, 38) respiratory deaths could be avoided.

**Conclusion:**

PM_2.5_ and O_3_ could significantly increase the risk of non-accidental and respiratory mortality in the middle-aged and elderly people in Lishui District of Nanjing. Exposed to air pollutants, men were more susceptible to O_3_ damage, and women were more susceptible to PM_2.5_ damage. Reduction of PM_2.5_ and O_3_ concentration in the air may have the potential to avoid considerable loss of lives.

## Background

With economic development, air pollution has become an important risk factor to people’s health. In 2019, air pollution ranked the fourth among the major risk factors of mortality in the world [1], causing 667 million deaths [2], comprising air pollutants such as particulate matter (PM) and ozone (O_3_). Since the Chinese government implemented China’s Action Plan of Prevention and Control of Air Pollution in 2013, concentration of PM_2.5_ (Particulate matter less than 2.5 μm in aerodynamic diameter) has dropped significantly [3]. However, along with the rapid development of urban infrastructure and growth in motor vehicle number, ozone concentration has increased dramatically in recent years [4, 5]. Nonetheless, Wang et al. showed that in spite of the decrease of PM_2.5_ concentration in 74 cities of China from 2013 to 2018, PM_2.5_ was still more harmful to human health than O_3_ [6]. A coordinated prevention and control of O_3_ and PM_2.5_ pollution is possibly the focus of improvement in air quality in China [7].

A study found that short-term exposure to air pollution, particularly PM_2.5_, was associated with an increased risk of hospitalization for multiple disease entities [8]. Long-term or short-term exposure to air pollution were widely reported to associate with increased all-cause, respiratory, and/or circulatory mortality [9–13]. In addition, studies have shown that exposure to air pollutants was associated with risk of out-of-hospital cardiac arrest, diabetes, cancer, and increased risk of death and cognitive decline [14–18]. Exposure to PM_2.5_ and O_3_, is not only harmful to human health but also associated with a substantial disease burden [19] and economic loss [20]. Maji etal showed that the proportion of all-cause, cardiovascular and respiratory premature deaths attributed to short-term environmental O_3_ exposure in China in 2019 increased by 19.6, 19.8, and 21.2% in comparison with those in 2015, with the most significant increase in the respiratory premature deaths [21]. Moreover, the pathophysiological mechanism of air pollution on human health has shown that the primary initiation pathway of regulating the effect of air pollution on human health, originates from the airways, including pollution-mediated oxidative stress, local inflammation, and ion channels or receptor activation. It is clear that air pollution bears the brunt of harm to the respiratory system [22]. Studies have shown that improving air quality can significantly reduce the risk of death due to exposure to air pollutants [23].

With increasing age, the physiological functions of the respiratory system and multiple organs would decline especially among the middle-aged and elderly people, possibly leading to slow-down immune responses and increased allergic reactions [24, 25]. Therefore older adults are the major susceptible population to air pollution [26, 27]. Considering PM_2.5_ and O_3_ are prominent local air pollutants, it is necessary to investigate the effects of short-term exposure to PM_2.5_ and O_3_ on non-accidental mortality and respiratory mortality among the middle-aged and elderly. Of all studies on air pollution and mortality conducted in China, focus on rural and semi-rural areas is rare. Allowing for the rapid development and urbanization in rural China, our study stands for an important starting point demonstrating the example of disease burden in relation to possible environmental pollution. Findings may inform preventative policies and countermeasures in other settings similar to Lishui District, a rural district undergoing substantial infrastructure development and growing of consumption pollution-intensive resources.

## Materials and methods

### Study area and population

Lishui District, one of the demonstration zone for Healthy China 2030, is located in the south of Nanjing, the capital city of Jiangsu Province. It has a northern subtropical monsoon climate with four distinct seasons, hot and humid in summer, cold and dry in winter. As of 2019, Lishui District has approximately 446,750 permanent residents, with an area of 1067 km^2^. We considered people aged 45 years and above as our study population.

### Study design

#### Data collection

The daily death records and the daily average concentration of atmospheric pollutants in Lishui District from January 1, 2015 to December 31, 2019, were obtained from the Lishui Smart City Operating Command Center of Nanjing, the official data integration and management center of Lishui District government, which collects selected administrative data from different agencies after the calibration and verification, and therefore data accuracy is substantially high. Specifically, mortality data was originally collected from the Lishui Bureau of Public Security, and the environmental data was originally collected from the Lishui Bureau of Environmental and Ecological Protection. These two government agencies run regular data quality checks and there was no missing data for the current study setting.

The daily death records included the mortality data of the permanent population in Lishui. Specific information included age, gender, date of birth and the underlying cause of death. We categorized the causes of death based on the ICD-10 (International Statistical Classification of Diseases and Related Health Problems 10th Revision) diagnosis, i.e., non-accidental mortality (A00-R99), and respiratory diseases (J00-J99). The environmental data included daily meteorological and atmospheric pollutants measures.

### Statistical analysis

We used the daily aggregated data from 2015 to 2019 to quantitatively assess the impact of PM_2.5_, and O_3_ exposure on non-accidental mortality and respiratory mortality. Daily mortality, air pollution, and meteorological data were described with average standard deviations and quartiles where appropriate. The relationship between air pollutants and meteorological conditions was evaluated using the spearman correlation. Mortality, air pollution, and meteorological data were linked by the date. Assuming that daily deaths in Lishui residents somewhat rare events and the correlation between explanatory variables and the number of deaths per day was mainly non-linear. Therefore, we constructed a generalized additive model (GAM) based on the Poisson distribution in which time-series analysis was used to establish the core model to estimate the association between mortality and air pollutant exposure. The model was specified as follows:

$$ \mathrm{Log}\left[\mathrm{E}\left({\mathrm{Y}}_{\mathrm{t}}\right)\right]=\upalpha +{\upbeta \mathrm{X}}_{\mathrm{t}}+\mathrm{ns}\left(\mathrm{Time},\mathrm{df}\right)+\mathrm{ns}\left({\mathrm{Z}}_{\mathrm{t}},\mathrm{df}\right)+\mathrm{DOW} $$

In this equation, t refers to the day of the observation; Y_t_ is the number of daily mortalities observed on day t; E(Y_t_) is the expected daily mortality rate on day t. α is the intercept; β represents the regression coefficient of the corresponding air pollutants; X_t_ represents the pollutant concentration on day t; Z_t_ represents the meteorological data on day t; DOW is a binary dummy variable; s is a non-linear variable with smoothing spline function. Previous studies have usually set the degrees of freedom (df) of time to 5 to 7 and meteorological factors to 3 to 6 [28–31]. The degree of freedom was selected according to the minimum value of the Akaike information criterion (AIC) of the Poisson model, and the smaller AIC value indicates the preferred model [32]. Considering the applicability and AIC value of the model, 6-df was used to adjust the time trend, seasonality, and temperature, whereas 3-df was used to adjust relative humidity in the model.

The lag effect of air pollutants on non-accidental mortality and respiratory mortality was modelled from the current day up to the 7th day (lag0-lag7). Previous studies have shown that cumulative effects may be underestimated by the single-day lag model [33]. Therefore, we further used the moving average of air pollutant concentrations from 2nd day to 8th day (lag01 to lag07) in the analysis. Considering that the decrease of PM_2.5_ might lead to the increase of photochemical flux and the acceleration of atmospheric oxidation, increasing of O_3_ concentration [34], we explored whether there is an interactive effect on the deaths arising from exposure to these two main pollutants in Lishui District by using the two-pollutant model to evaluate the confounding effect of pollutants. After establishing the statistical models that includes all control variables and checking the applicability, we separately included air pollutants into the model. Additional analyses were carried out stratified by gender (female and male), age group (45–64 years, 65–84 years, 85 years or older), or season (spring, summer, autumn, winter). Results were expressed as excess risk (ER) and 95% confidence intervals (CI) of daily deaths associated with 10 μg/m^3^ increase in pollutants’ concentration.

We further estimated the death burden attributable to short-term exposure to O_3_ and PM_2.5._ The counts of different death outcomes attributable to air pollutants were estimated using: AC_ij_ = N_ij_ *(RR_ij_ − 1)/ RR_ij_, where RR_ij_ is the relative risk for disease j at lagi based on the relative risk functions. N_ij_ is the death number of disease j at lagi. AC_ij_ is the attributable counts of disease j at lagi. We then calculated the total attributable counts of disease j (AC_j_) by summing the AC_ij_ of the study period. Finally, the population attributable fractions (PAF) were calculated by dividing the total AC_j_ by the total number of deaths in the middle-aged and elderly people. All Statistical analysis was performed using R software, version 4.0.3. The statistical significance of all analyses was set as *P* < 0.05.

## Result

**Fig. 1:**
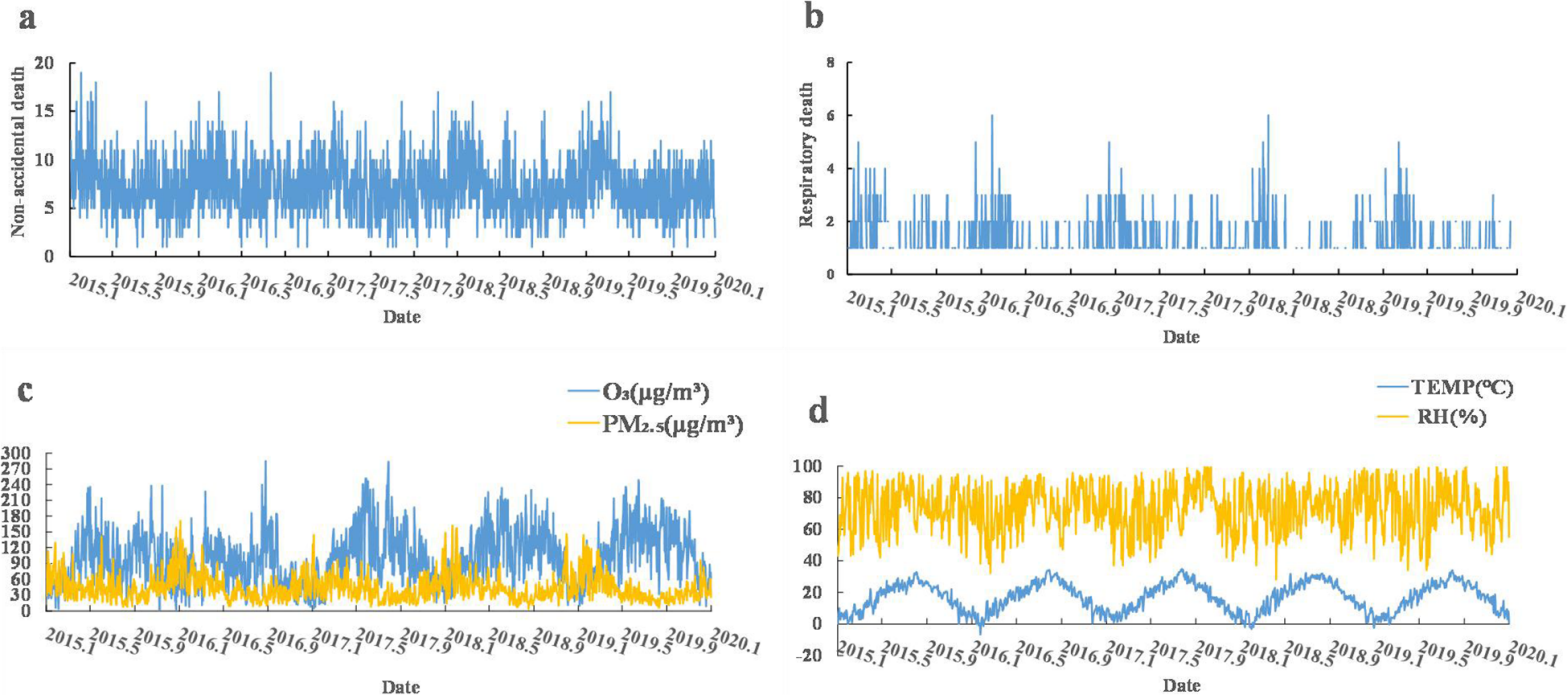
Time-series of pollutants, meteorological factors and daily death counts in Lishui district from 2015 to 2019. **a** Time-series of non-accidental mortality; **b** Time-series of respiratory mortality; **c** Time-series of O_3_ and PM_2.5_; **d** Time-series of temperature and relative humidity

**Fig. 2:**
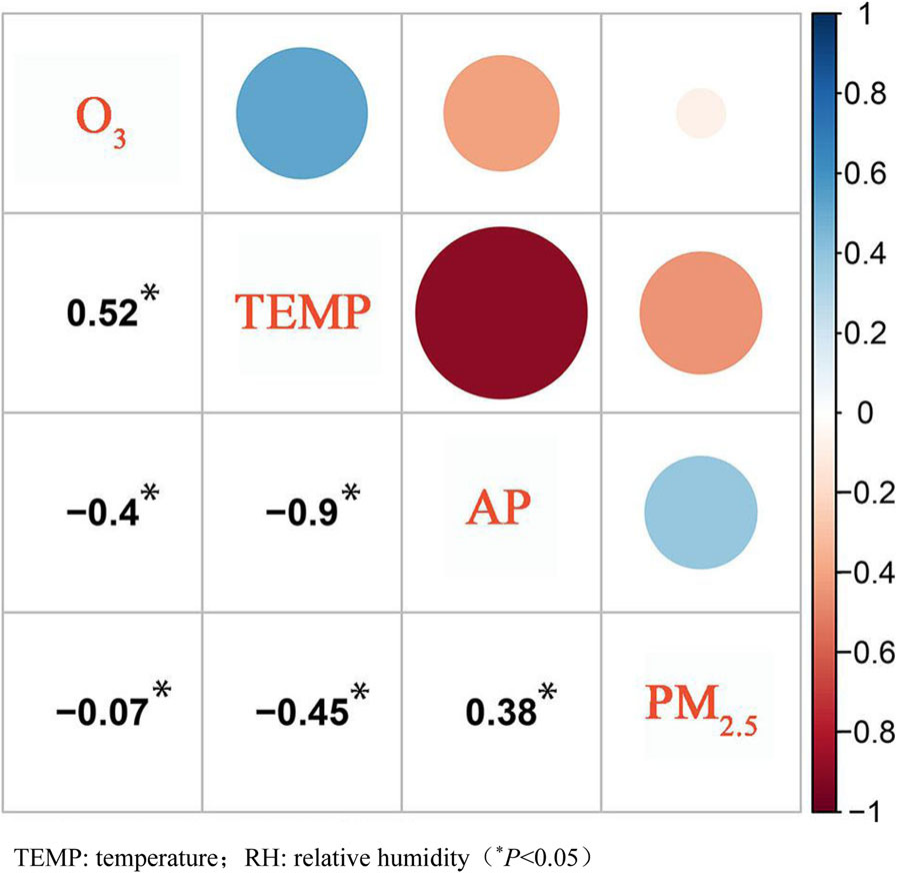
Spearman correlation coefficients between daily air pollutants and meteorological parameters

Table 1 shows the descriptive summary for daily mortality, air pollutants, and meteorological data in Lishui District of Nanjing during the period of 2015–2019. The total number of non-accidental mortality and respiratory mortality among the middle-aged and elderly (≥45 years) in Lishui District was 13,160 and 1478 respectively. A seasonal pattern of daily mortality was also observed, with higher mortality in winter (Fig. 1). The daily average temperature was 16.90 °C (Range: -6.70 °C, 34.70 °C), the daily relative humidity readings were measured in integers with an average of 72.99% (Range: 28, 100%). The 24-h PM_2.5_ concentrations were measured in integers with an average of 43.57 μg/m^3^ (Range: 26 μg/m^3^, 171 μg/m^3^). The maximum daily 8-h concentrations of O_3_ (MDA8 O_3_) were measured in integers with an average of 100.13 μg/m^3^ (Range: 2 μg/m^3^, 285 μg/m^3^). O_3_ concentration was to a moderate degree positively correlated with average temperature (*r* = 0.52, *P* < 0.05), was to a moderate degree positively correlated with the relative humidity (*r* = − 0.38, *P* < 0.05), and was slightly negatively correlated with PM_2.5_ concentration. PM_2.5_ was moderately correlated with the temperature (*r* = − 0.45, *P* < 0.05), and was slightly negatively correlated with the relative humidity (Fig. 2).

**Table 1:** Daily deaths, air pollutants, and meteorological factors in Lishui district, 2015–2019

Variables	Mean	SD	Min	P50	Max
Daily mortality counts					
Non-accidental mortality	7.21	2.90	0	7	19
Respiratory mortality	0.81	0.96	0	1	6
Gender (n)					
Male	4.10	2.07	0	4	13
Female	3.11	1.86	0	3	11
Age group (n)					
45–64 years	1.60	0.82	1	1	6
65–84 years	3.77	1.73	1	4	12
85 years or older	2.08	1.09	1	2	7
PM_2.5_ (μg/m^3^)					
All year	43.57	25.05	4	38	171
Spring	45.26	18.63	9	43	125
Summer	29.25	15.65	4	26	141
Autumn	36.84	18.19	10	34	146
Winter	63.26	30.98	14	56	171
O_3_ (μg/m^3^)					
All year	100.13	50.58	2	93.60	285.00
Spring	117.68	45.36	11	113.00	252.00
Summer	120.90	52.66	10	118.00	285.00
Autumn	101.25	46.11	2	99.00	238.00
Winter	59.90	30.39	2	59.00	176.00
Average temperature (°C)					
All year	16.91	9.07	−6.70	17.80	34.70
Spring	16.64	5.54	2.90	17.20	31.70
Summer	27.36	3.28	18.70	27.20	34.70
Autumn	18.07	5.65	0.10	17.90	29.30
Winter	5.36	3.50	-6.70	5.30	14.90
Relative humidity (%)					
All year	72.99	13.99	28	73	100
Spring	68.46	15.09	28	68	100
Summer	76.95	10.70	49	77	100
Autumn	75.20	12.77	42	75	100
Winter	71.33	15.33	32	71	100

Notes: Seasons were separated into Spring (Mar-May), Summer (Jun-August), Autumn (Sept-Nov) and Winter (Dec-Feb)

**Fig. 3:**
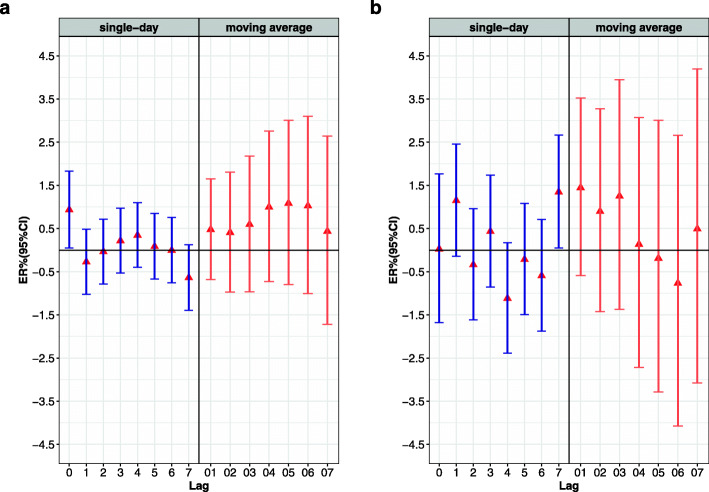
The ER (95% CI) associated with 10 μg/m^3^ increase of mortality; **a** PM_2.5_ led to non-accidental mortality; **b** O_3_ led to respiratory mortality

After adjusting for the time, day of the week, and weather conditions, we evaluated the single-day lag effect (lag0-lag7) and multi-day moving average lag effect (lag01-lag07) on non-accidental mortality and respiratory mortality (Fig. 3). For every increase in PM_2.5_ concentrations by 10 μg/m^3^, the greatest excessive risk of non-accidental mortality on the current day (lag0) increased by 0.94% (95% CI: 0.05, 1.83%), and at lag7 the excessive risk of respiratory mortality increased by 0.57% (95% CI: − 1.53, 2.72%). For every increase in O_3_ concentration by 10 μg/m^3^, on the current day (lag0) the excessive risk of non-accidental mortality increased by 0.10% (95% CI: − 0.46, 0.67%), and the greatest excessive risk of respiratory mortality at lag7 increased by 1.35% (95% CI: 0.05, 2.66%). The increase of PM_2.5_ and O_3_ concentration had no statistical significance on the moving average lag effects of non-accidental mortality and respiratory mortality. To avoid multiple collinearities, only the two-pollutant model was used to detect the robustness of the model, and the multi-pollutant model was not considered. Compared with the single pollutant model, the results of the two-pollutant model had no significant change, and therefore the current model was somewhat robust (Table 2)

**Table 2:** The excess risk (95% CI) of daily mortality associated with 10 μg/m^3^ increase

Variables	Non-accidental mortality	Respiratory mortality
**PM**_**2.5**_		
Single pollutant model	0.9363% (0.0492, 1.8312%)^*^	0.6029% (−1.5060, 2.7571%)
**+** O_3_	0.9359% (0.0366, 1.8434%)^*^	0.6026% (−1.5069, 2.7572%)
**O**_**3**_		
Single pollutant model	0.1501% (−0.2682, 0.5701%)	1.3469% (0.0479, 2.6627%)^*^
**+** PM_2.5_	0.1460% (− 0.2722, 0.5659%)	1.3384% (0.0363, 2.6574%)^*^

Note. ^*^P < 0.05

**Fig. 4:**
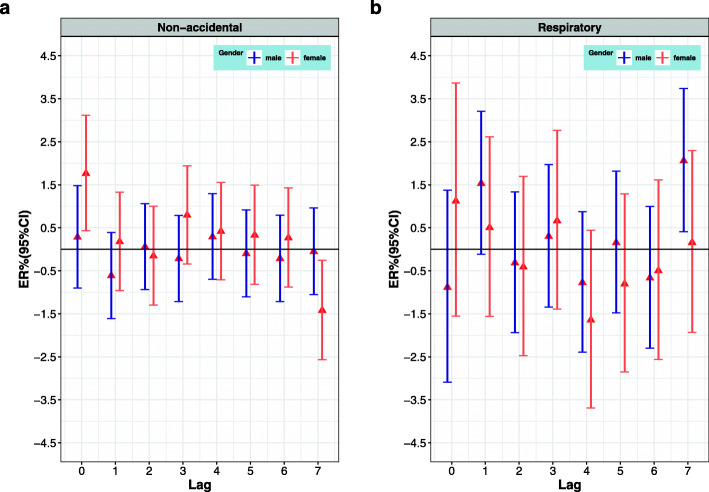
The ER (95% CI) in gender lag-response relationship associated with 10 μg/m^3^ increase of mortality; **a** PM_2.5_ led to non-accidental mortality; **b** O_3_ led to respiratory mortality

**Fig. 5:**
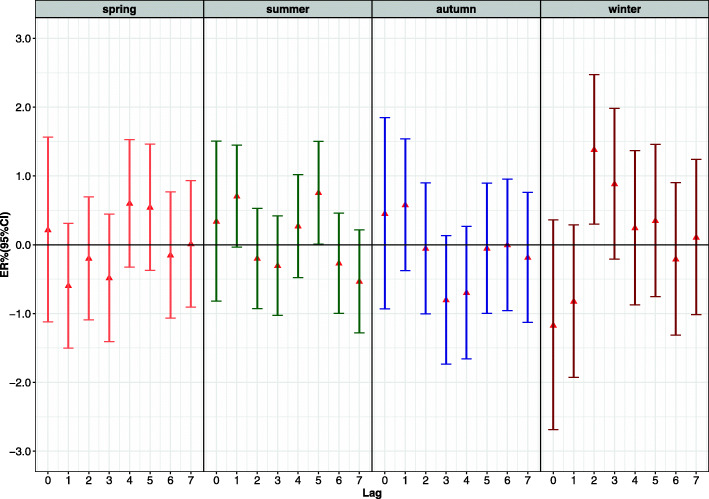
The ER (95% CI) in season lag-response relationship associated with 10 μg/m^3^ increase of O_3_ led to non-accidental mortality

Tables 3 and 4 show the effect modification, after stratifying daily mortality by age, sex, and season. Figure 4 (a) shows that the single pollutant model, for every 10 μg/m^3^ increase in PM_2.5_, the greatest excessive risk of non-accidental mortality among middle-aged and elderly women on the current day (lag0) increased by 1.77% (95% CI: 0.43, 3.12%). There was no statistically significant difference in the effect of PM_2.5_ on male non-accidental mortality (*P* < 0.05). Figure 4 (b) shows that in every 10 μg/m^3^ increase in O_3_ led to 1.38% (95% CI: 0.30, 2.47%) increase in respiratory mortality at lag7. And the effect of women was no statistical significance. Figure 5 shows that for every 10 μg/m^3^ increase in O_3_, non-accidental mortality in summer and winter increased by 0.75% (95% CI: 0.01, 1.50%) and 1.38% (0.30, 2.47%) at lag2 and lag5 respectively. The effect of O_3_ on non-accidental mortality was not statistically significant in spring and autumn (*P* > 0.05). The increase of PM_2.5_ and O_3_ concentrations has different maximum lag effects in different age groups, but the effect is not statistically significant.

**Table 3:** The non-accidental maximum ER (95% CI) in lag days, stratified by age, sex and season

Variables	Non-accidental deaths			
	Lag	PM_2.5_	Lag	O_3_
All	Lag0	0.94% (0.05, 1.83%)^*^	Lag1	0.15% (− 0.27, 0.57%)
Age(yeas)				
45–64	Lag0	0.99% (− 1.34, 3.37%)	Lag1	0.31% (− 0.92, 1.54%)
65–84	Lag5	1.04% (−2.30, 4.51%)	Lag7	0.25% (− 0.40, 0.90%)
85+	Lag0	1.13% (− 0.63, 2.91%)	Lag0	0.52% (− 0.63, 1.68%)
Sex				
Male	Lag4	0.29% (− 0.70, 1.29%)	Lag1	0.16% (− 0.40, 0.71%)
Female	Lag0	1.77% (0.43, 3.12%)^*^	Lag0	0.67% (−0.18, 1.53%)
Season				
Spring	Lag0	1.12% (−1.43, 3.72%)	Lag4	0.60% (−0.33, 1.53%)
Summer	Lag5	2.38% (−0.11, 4.94%)	Lag5	0.75% (0.01, 1.50%)^*^
Autumn	Lag3	1.09% (−1.02, 3.24%)	Lag0	0.45% (−0.93, 1.85%)
Winter	Lag0	0.96% (−0.38, 2.33%)	Lag2	1.38% (0.30, 2.47%)^*^

Note. ^*^P < 0.05

**Table 4:** The respiratory maximum ER (95% CI) in lag days, stratified by age, sex and season

Variables	Respiratory deaths			
	Lag	PM_2.5_	Lag	O_3_
All	Lag2	0.60% (− 1.51, 2.76%)	Lag7	1.35% (0.05, 2.66%)^*^
Age(year)				
45–64	Lag0	0.39% (−15.11, 18.71%)	Lag4	0.39% (− 11.35, 13.67%)
65–84	Lag5	1.04% (−2.30, 4.51%)	Lag5	0.69% (− 1.36, 2.79%)
85+	Lag0	0.59% (−3.27, 4.60%)	Lag4	0.11% (−2.21, 2.48%)
Sex				
Male	Lag4	1.03% (−1.65, 3.78%)	Lag7	2.06% (0.41, 3.74%)^*^
Female	Lag0	2.21% (−1.73, 6.30%)	Lag0	1.12% (−1.55, 3.87%)
Season				
Spring	Lag0	2.49% (−4.92, 10.47%)	Lag5	1.50% (−1.41, 4.50%)
Summer	Lag6	7.76% (−0.11, 16.25%)	Lag3	1.19% (−1.14, 3.58%)
Autumn	Lag6	3.26% (−3.81, 10.85%)	Lag6	0.14% (−2.93, 3.31%)
Winter	Lag7	1.97% (−0.87, 4.90%)	Lag4	2.16% (−0.87, 5.29%)

Note. ^*^P < 0.05

Table 5 shows the numbers and fractions of non-accidental mortality and respiratory mortality attributable to air pollutants among the middle-aged and elderly in Lishui district. The Population Attributable Fractions (PAF) of non-accidental mortality were 0.84% (95% CI: 0.00, 1.63%) for PM_2.5_ and the PAF of respiratory mortality were 0.14% (95% CI: 0.05, 0.26%) for O_3_. Every 10 μg/m^3^ decrease in PM_2.5_ could save 122 (95% CI: 6, 237) people from non-accidental deaths, and every 10 μg/m^3^ decrease in O_3_ could save 10 (95% CI: 1, 38) people from respiratory deaths.

**Table 5:** PAC (95% CI) and PAF (95% CI) in association with air pollutants in 2015–2019

	Pollutants	PAC (95% CI)	PAF (95% CI)
Non-accidental mortality	PM_2.5_	122 (6, 237)	0.84% (0.00, 1.63%)
Respiratory mortality	O_3_	20 (1, 38)	0.14% (0.05, 0.26%)

Note: PAC: Population Attributable Counts; PAF: Population Attributable Fractions

## Discussion

This study used a time-series model to investigate the relationship between exposure of air pollutants (PM_2.5_ and O_3_) and non-accidental mortality and respiratory mortality in Lishui District of Nanjing, Jiangsu Province, China from 2015 to 2019. Results showed that short-term exposure to PM_2.5_ and O_3_ was positively correlated with an increased risk of non-accidental and respiratory mortality. The daily average concentration of PM_2.5_ was 43.57 μg/m^3^, which was higher than the National Ambient Air Quality Standard (NAAQS) first-level standard, but lower than the second-level standard (the first-level standard is 35 μg/m^3^, the second-level standard is 75 μg /m^3^). The MDA8 O_3_ was 100.13 μg/m^3^, which was also higher than the NAAQS first-level standard, but lower than the second-level standard (the first-level standard is 100 μg/m^3^ and the second-level standard is 160 μg/m^3^). The seasonal fluctuation of air pollution demonstrated that PM_2.5_ concentrations were higher in spring and winter than in summer and autumn and reached its peak in summer, whereas O_3_ concentrations were higher in summer and autumn than in spring and winter, and peaked in winter. A seasonal pattern in the number of daily deaths was also observed, with higher mortality in winter. This observed seasonal fluctuation may be related to the increase in pollutants. For example, industrial activities and combustion emissions in winter are more frequent to produce more PM_2.5_ [35], whereas high temperature and sufficient sunshine in summer are favorable conditions for photochemical reaction to produce O_3_ [36]. Using chemical industrial solvents and emitting the volatile organic compounds and nitrogen oxides from automobile exhaust may cause high levels of O_3_ as well [37].

We found that in the single pollutant model, PM_2.5_ demonstrated acute effects on non-accidental mortality. Every 10 μg/m^3^ increase in PM_2.5_ was associated with a 0.94% (95% CI: 0.05, 1.83%) increase in non-accidental mortality at lag0. A study conducted in a highly polluted area in China found that 10 μg/m^3^ increase in PM_2.5_ was associated with 0.36% (95% CI: 0.10, 0.63%) increase of non-accidental mortality [38]. Lin et al. found that every 10 μg/m^3^ increase in PM_2.5_ was associated with 1.50% (95% CI: 0.50–2.50%) of non-accidental mortality among the elderly aged over 65 years [39]. A study conducted in 75 cities in the United States showed that for every 10 μg/m^3^ increase in PM_2.5_, the non-accidental mortality rate increased by 1.18% (95% CI: 0.93, 1.44%) [40]. Another large-scale study involving multiple countries and regions found that for every 10 μg/m^3^ increase in PM_2.5_, the daily non-accidental mortality rate increased by 0.68% (95% CI: 0.59, 0.77%) [41]. Although our results showed that the impact of PM_2.5_ on non-accidental mortality in Lishui District was slightly higher, it was generally consistent with the results of previous research reports in China. This difference may be mainly related to the difference with study settings, for example the age difference of the exposed population. Moreover, the sources and chemical composition of PM_2.5_ in different regions are different, which may also lead to different effects on mortality.

We also found that O_3_ had acute effects on respiratory mortality. Every 10 μg/m^3^ increase in O_3_ was associated with an increase in respiratory disease mortality by 1.35% (95% CI: 0.05, 2.66%) at lag7. A study in Jinan showed that Every 10 μg/m^3^ increase in O_3_ was associated with a 0.98% (95% CI: 0.46, 1.49%) increase in respiratory mortality at lag3 [42]. Another study in Hefei showed that every 10 μg/m^3^ increase in O_3_ led to a 2.22% (95% CI: 0.56, 3.90%) increase in respiratory mortality [38]. A Sichuan study found that every 10 μg/m^3^ increase in O_3_ led to a 0.78% (95% CI: 0.12, 1.44%) increase in respiratory mortality [43]. Our finding was consistent with the previous results [44–46]. With the rapid development of the economic level and the acceleration of the urbanization process, the production of industrial manufacturing was also increasing, which may lead to the increase of volatile organic compounds (VOCs) emissions [47]. This may be one of the reasons that O_3_ in Lishui District had a greater impact on the respiratory mortality in the middle-aged and elderly population. In this study, the impact of multi-day moving average lag was higher than that of single-day lag, but the effect was not statistically significant, which was also consistent with the previous results [48].

Subgroup analysis showed that air pollutants were significantly related to non-accidental and respiratory mortality in different genders and seasons. Women were more susceptible to PM_2.5_ in terms of non-accidental mortality. This was consistent with the Shin et al. study [49] and Hu et al. study [50]. Women may have stronger airway responsiveness in addition to hormones or other factors, and therefore women might have a stronger physiological response to air pollutants [51, 52]. However, there was also conflicting evidence that men were more susceptible to the impact of PM_2.5_ on non-accidental mortality [53, 54]. In contrast, we found that men were more susceptible to the effects of O_3_ on respiratory mortality than women. A research carried out in Shenzhen also found the similar result [55], which could be explained by the fact that pneumonia and bronchitis were more commonly observed in men who had a smoking history and different occupational exposures, which may exacerbate the impact of O_3_ on respiratory mortality [56].

The O_3_ concentration in summer had a statistically significant effect on non-accidental mortality. This is consistent with the finding of Zanobetti et al. [57], allowing for that in summer the ozone precursor substances in the air produce O_3_ faster as the temperature rises [58]. We also found that although the concentration of O_3_ in winter was at the lowest level throughout the year, the effect of O_3_ on non-accidental mortality was also substantial. A study in Nanjing found that the concentration of indoor O_3_ in winter may be greater than that of outdoor O_3_ [59], and therefore impact of O_3_ exposure on excess deaths might be underestimated in the current study. Research conducted in East Asia found that O_3_ levels in different seasons have varying degrees of impact on non-accidental mortality [60], which indicates potential geographical heterogeneity [61]. To identify susceptible groups, we also explored the potential modification effects of age, but in our study, we did not observe significant modification effects of age groups.

In the attributable fraction analysis, nearly 0.84% of non-accidental mortality can be attributable to PM_2.5_, and reduction in the concentration of PM_2.5_ could save 122 (95% CI: 6, 237) lives of the middle-aged and elderly people. In addition, 0.14% respiratory mortality can be attributable to O_3_, and reduction in the concentration of O_3_ could save 20 (95% CI: 1, 38) lives of middle-aged and elderly people. This finding highlights the gain in population health and reduction in disease burden in association with air pollution. Therefore, relevant authorities in Lishui District might take measures to improve the quality of atmospheric environment to enhance population health.

Considering the impact on respiratory mortality as perhaps the most direct effect caused by environmental pollutants during the contact with airways, our study selected the most prominent atmospheric pollutants PM_2.5_ and O_3_ in Lishui District and analyzed their relationship with the mortality of the study population. Previous studies on respiratory mortality in relation to atmospheric pollutants demonstrated inconsistent findings. For example, ozone was found having little impact on the non-accidental deaths in Hefei, capital city of Anhui province in China [62]. Our findings add to the literature by providing a new evidence of the relationship between respiratory mortality and atmospheric pollutants PM_2.5_ and O_3_.

This study has some limitations. First, we used the average concentration of air pollutants recorded at the monitoring sites as the population exposure level, without considering the indoor exposure. This would lead to exposure measurement errors and deviations in the accuracy and intensity of risk estimates. Secondly, the daily mean death number due to respiratory diseases might be too low to draw a safe conclusion. The results cannot be extrapolated to the entire Nanjing or other regions of China, and therefore results should be interpreted with caution. Moreover, this study did not collect information on smoking history, body mass index, drug history, and educational level. These potential confounding factors may also have a latent impact on the association between air pollution and mortality. In the two-pollutant model, the effect of each pollutant on non-accidental and respiratory mortality was reduced, which was inconsistent with previous studies [38, 62]. Considering concentrations of PM_2.5_ and O_3_ varied in an opposite way by seasons, for example, higher PM_2.5_ concentrations in winter but higher O_3_ concentrations in summer, a possible offset function would explain the observed inconsistency. Nonetheless, future studies would be carried out to investigate the joint effects of these two pollutants.

## Conclusion

This study shows that among the middle-aged and elderly residents in Lishui District of Nanjing, China, short-term exposure to PM_2.5_ and O_3_ would increase the risk of non-accidental death and respiratory death. Our findings complement previous studies by revealing that air pollutants have a lag effect on the health of the population in rural areas undergoing rapid socioeconomic development. These findings call for new initiatives including implementation of more stringent air pollutant emission control policies to improve population health.

## Data Availability

The datasets generated and/or analyzed during the current study are not publicly available due to the sensitive nature of the raw data and restrictions apply to the availability of the raw data.

## Abbreviations

O_3_: Ozone
PM: Particulate matter
PM_2.5_: Particulate matter less than 2.5 μm in aerodynamic diameter
CI: Confidence intervals
MDA8 O_3_: Maximum daily maximum 8-h average concentration
TEMP: temperature
RH: relative humidity
NAAQS: National Ambient Air Quality Standard
ICD-10: International Statistical Classification of Diseases and Related Health Problems 10th Revision
GAM: Generalized additive model
ER: Excess risk
df: degrees of freedom
AIC: Akaike information criterion
PAF: Population attributable fractions
PAC: Population Attributable Counts
